# Quantitative Measurements of Codeine and Fentanyl on a Surface-Enhanced Raman-Active Pad

**DOI:** 10.3390/molecules24142578

**Published:** 2019-07-16

**Authors:** Chetan Shende, Amelia Farquharson, Carl Brouillette, Wayne Smith, Stuart Farquharson

**Affiliations:** Real-Time Analyzers, Inc., Middletown, CT 06457, USA

**Keywords:** SERS, Raman, opioid detection, codeine, fentanyl, forensics

## Abstract

The USA is in the midst of an opioid crisis that included over 60,000 overdose fatalities in 2017, mostly unintentional. This is due to excessive use of prescription opioids and the use of very strong synthetic opioids, such as fentanyl, mixed with illicit street drugs. The ability to rapidly determine if people or packages entering the country have or contain drugs could reduce their availability, and thereby decrease the use of illicit drugs. In an effort to address this problem, we have been investigating the ability of surface-enhanced Raman spectroscopy to detect trace amounts of opioids on clothing and packages. Here, we report the measurement of codeine and fentanyl at 100 ng/mL for 5 min on a pad impregnated with gold colloids, as well as a preliminary measurement of 500 pg of fentanyl on a glass surface using one of these pads. The calculated limit of detection for this measurement was 40 pg. This data strongly suggests that these pads, used with portable Raman analyzers, would be invaluable to airport security, drug raids, crime scenes, and forensic analysis.

## 1. Introduction

The opioid epidemic consists of two very different situations that ultimately lead to fatalities. The overuse of prescription opioids like codeine led to ~17,000, primarily unintentional, overdose deaths in the USA in 2017 [1], while the massive illegal influx of fentanyl into the USA led to ~20,000 overdose deaths in 2016, mostly of addicts using drugs laced with fentanyl [1,2]. Codeine is often prescribed in combination with nonsteroidal anti-inflammatory drugs, such as acetaminophen, aspirin, and ibuprofen, to treat varying degrees of pain, and was the most common drug used in the world in 2013 [3]. The extreme potency of fentanyl, ~100 times that of morphine [4], makes it ideal for smuggling across borders, as only a small amount represents a substantial payout. For example, 250 pounds were seized at the Mexican border in January 2019 [5], and was valued at ~$3.5 million with the potential of killing >50 million people [6]. Many of the deaths could be prevented if these drugs could be rapidly detected on people’s fingers, clothing, and packages at borders and airports so that the drugs are seized and never enter the illicit drug market. 

In recent years, Raman spectroscopy has become a relatively common tool used by a number of agencies to identify powders suspected of being biological agents [7], explosives [8,9], or drugs [10,11,12,13]. However, the sensitivity of Raman spectroscopy is insufficient to detect nanogram amounts of drugs on surfaces that are essentially invisible. We believe that surface-enhanced Raman spectroscopy (SERS) is ideally suited to meet this need. SERS employs the interaction of chemicals with the plasmon field generated by lasers at the surface of gold or silver nanoparticles to enhance the Raman signal intensity by as much as six orders-of-magnitude [14,15]. Adding these metal nanoparticles to chemicals, such as drugs, allows nanogram detection. The application of SERS to illicit drug detection began at the turn of the century and included cocaine [16], barbiturates [17], and amphetamines [18,19,20]. More recently, several publications have described the use of hand-held Raman spectrometers for field measurements [21,22,23,24]. 

Here we describe the development of a SERS-active pad that is suitable for swabbing surfaces and its application to the measurement of codeine and fentanyl. In addition to their role in the opioid epidemic, these two drugs were also chosen as they represent two different structural classes of opiates. Codeine, a natural drug extracted from opium, represents the traditional structure of opioids, while fentanyl, a fully synthetic opioid, represents the latest in designer drugs that are far more potent in affecting the opioid receptors. 

## 2. Results and Discussion

**Figure 1:**
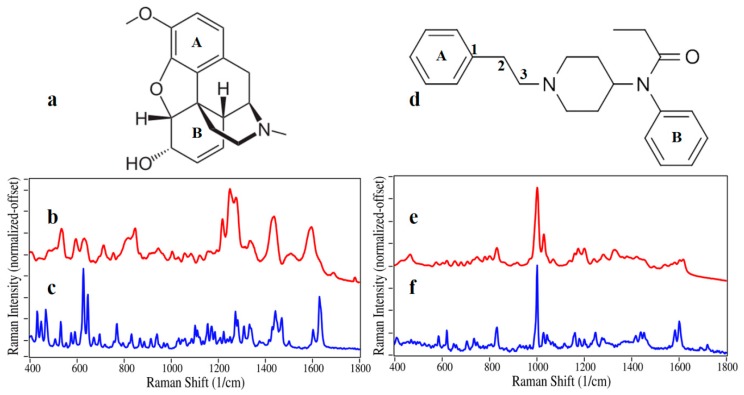
Codeine (**a**) chemical structure, (**b**) surface-enhanced Raman spectra (SERS), and (**c**) Raman spectra. Fentanyl (**d**) chemical structure, (**e**) SERS, and (**f**) Raman spectra. SERS conditions: 50 µg/mL, 40 mW at 785 nm, 3-sec scan per spectrum. Raman spectra are from Nicolet spectral library that used ~500 mW at 1064 nm.

The SERS for codeine is considerably different from its Raman spectrum (Figure 1a–c), most likely due to the attraction of the hydroxyl hydrogen to the electronegative gold affecting the orientation of the drug on the metal surface and its interaction with the plasmon field, which greatly affects the relative enhancement of the various vibrational modes. The SERS of codeine is dominated by peaks at 535, 595, 630, 1220, 1250, 1275, 1340, 1435, and 1595 cm^−1^, and based on its Raman spectrum [25], these peaks are assigned to A- plus B-ring CH out-of-plane bending, B-ring CC bending, B-ring CC bending, A-ring CH bending, general CH bending, general CCC bending, ring CH bending, CH_3_ bending, and B-ring CC stretching, respectively. In contrast, the SERS of fentanyl is very similar to the Raman spectrum (Figure 1d–f), as there appears to be no preferred orientation to the gold surface. Both spectra are dominated by the intense 1000 cm^−1^ peak and a weaker peak at 1030 cm^−1^ due to the A- and B-ring phenyl CCC trigonal bends, respectively [26]. Other minor peaks occur at 830, 1165, 1180, 1200, 1330, 1605, and 1620 cm^−1^, which are assigned to A-ring out-of-plane asymmetric CH bending, B and A-phenyl CC stretching, C1–C2 symmetric stretching, piperidine CH wagging, and B and A-phenyl CC symmetric stretching, respectively. 

**Figure 2:**
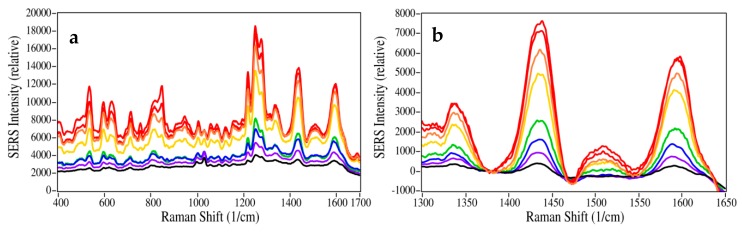
SERS of codeine at 50, 25, 10, 5, 1, 0.5, 0.25, and 0.1 µg/mL. (**a**) Full spectral view as measured, and (**b**) expanded view of the 1435 cm^−1^ peak, with the baseline set to 0 at 1380 cm^−1^. Conditions: 40 mW at 785 nm, 3-sec scan per spectrum.

**Figure 3:**
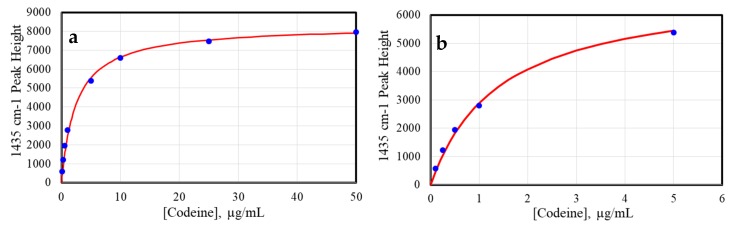
Plot of codeine 1435 cm^−1^ peak height as a function of the concentration, fit with a Langmuir curve for (**a**) 100 ng/mL to 50 µg/mL, and (**b**) 100 ng/mL to 5 µg/mL.

The SERS pads were tested by measuring codeine and fentanyl from 100 ng/mL (100 ppb) to 50 µg/mL (50 ppm) using the spectral conditions described in the Materials and Methods below. In each case, 20 µL of sample was deposited on the pad, allowed to dry for 5 min, placed in the spectrometer sample compartment, and measured (Figure 2). The intensities of the codeine spectra were plotted as a function of sample concentration (Figure 3 using the 1435 cm^−1^ peak, with the baseline at 1380 cm^−1^ set to zero (Figure 2b). The spectral intensity is a function of the available nanoparticle surface area, and can be approximated using the Langmuir isotherm equation [27]:

Θ = [kC/(1 + kC)]S(1)

where Θ is the surface coverage (here expressed as the SERS peak height), k is a constant, C is the sample concentration, and S is a scaling constant added to produce the correct SERS peak height. The gold nanoparticles were virtually covered at ~25 µg/mL codeine (Figure 3a). 

**Figure 4:**
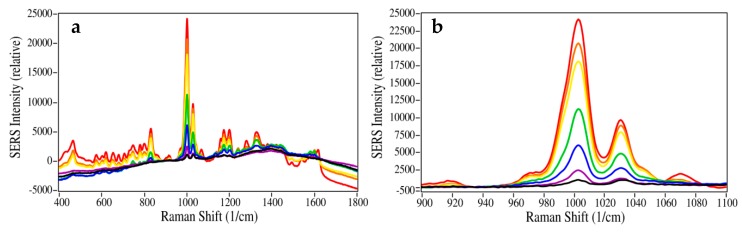
SERS of fentanyl at 50, 25, 10, 5, 1, 0.5, and 0.1 µg/mL. (**a**) Full spectral view as measured, and (**b**) expanded view of the 1000 cm^−1^ peak. The baselines for both spectral sets were set to 0 at 940 cm^−1^. Conditions: 40 mW at 785 nm, 3-sec scan per spectrum.

**Figure 5:**
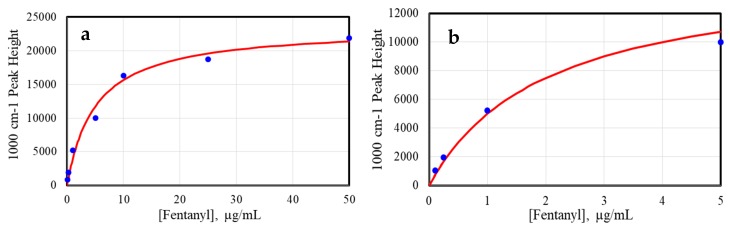
Plot of fentanyl 1000 cm^−1^ peak height as a function of the concentration, fit with a Langmuir curve for (**a**) 100 ng/mL to 50 µg/mL, and (**b**) 100 ng/mL to 5 µg/mL.

The intensities of the fentanyl spectra (Figure 4) were plotted as a function of the sample concentration (Figure 5) using the 1000 cm^−1^ peak, with the baseline at 940 cm^−1^ set to zero (Figure 4b). Again, the SERS intensities as a function of concentration followed the standard Langmuir equation. As with codeine, the gold nanoparticles were virtually covered at ~25 µg/mL fentanyl (Figure 5a).

The lowest measured spectra of 100 ng/mL for each drug were used to calculate a limit of detection (LOD) based on a signal-to-noise ratio (S/N) of 3. For each spectrum, the baseline corrected peak height (see Figure 7a inset) was divided by the standard deviation noise for a spectral segment between 1900 and 2000 cm^−1^. The spectral width was chosen to match that of the codeine 1435 cm^−1^ and fentanyl 1000 cm^−1^ peaks, and the spectral region was chosen where there is only detector noise. The LODs were 5 ng/mL (100/[593/9]/3) and 6 ng/mL (100/[2584/50]/3) for codeine and fentanyl, respectively. 

**Figure 6:**
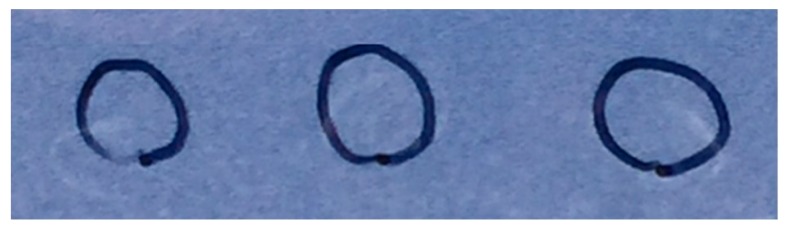
Photograph of three 10 µL drops of 50 ng/mL fentanyl dried on part of a glass slide. This concentration corresponds to 500 pg. The samples, deposited on the opposite side of the ~5 mm diameter black marked rings, migrated during evaporation, forming nearly invisible white rings.

A preliminary surface measurement was performed, as might be done at airport security or during a drug raid. Three samples each of 50 and 100 ng fentanyl per mL water were deposited on a glass slide and allowed to air dry, producing nearly invisible 0.5 and 1 ng white ring residues, respectively (Figure 6 and Figure S1). 

**Figure 7:**
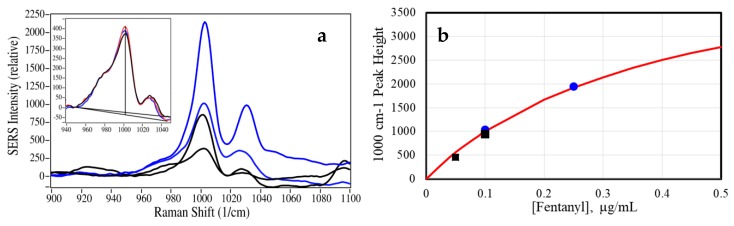
(**a**) SERS of 50 and 100 ng/mL fentanyl from glass surface (black), and 100 and 250 ng/mL fentanyl reference spectra (blue). Inset: Three individual 50 ng/mL sample spectra (note baseline-corrected heights). (**b**) Concentration plot showing corresponding peak heights (black squares, baseline tilt corrected) with reference samples (blue circles). Conditions: 40 mW at 785 nm, 3-sec scan per sample, three spectra averaged (except inset). No spectral smoothing was necessary.

Then, 10 µL methanol was added to a pad, which was swabbed across a pre-marked area on the opposite side of the glass slide to collect the sample. The pad was allowed to dry for 5 min prior to the SERS measurements. The three measured spectra for the 50 and 100 ng samples were then averaged (Figure 7a), and the peak heights were used to calculate the amount fentanyl collected and the effectiveness of the pads. The baseline-corrected 1000 cm^−1^ peak heights of 412 and 943 were used with the Equation (1) calibration curve to calculate collected concentrations of 36 and 93 ng/mL, respectively (Figure 7b). The standard deviation for the three 50 ng/mL samples was ±4 ng/mL. The collection efficiencies were 72% and 93%, respectively. These results are not surprising, as the lower concentration would have covered less of the pad. While the averaged 50 ng/mL spectral S/N suggests a LOD of 4 ng/mL (50/(36/3), similar to the calibration data LOD, no spectrum was obtained for a 10 ng/mL sample. It is assumed that the sample was unevenly spread over the pad surface, and could potentially have been detected using a scanning system. Finally, it is worth noting that the 10 µL of the 50 ng/mL samples dried on the surface each represented only 500 pg and a calculated LOD of 40 pg (see Supplemental).

## 3. Materials and Methods

Analytical-grade phosphate-buffered saline (PBS), sodium citrate, chloroauric acid, cetyltrimethylammonium bromide, and HPLC-grade water were all purchased from Sigma Aldrich (St Louis, MO, USA). Glass fiber sheets were purchased from Sigma Millipore (Burlington, MA, USA), cut to size, and used as the support structure for the SERS-active pads. Forensic-grade codeine and fentanyl (1 mg/mL methanol and acetonitrile, respectively) were purchased from Cerilliant Corp (Round Rock, TX, USA). Samples were prepared by adding 10 µL of the forensic drug to 90 µL HPLC water to produce 100 µg/mL samples. These samples were further diluted with water to produce 50, 25, 10, 5, 1, 0.5, 0.25, and 0.1 µg/mL samples for additional measurements. In addition to these samples, a 0.05 fentanyl sample was prepared and used for the preliminary surface measurements. 

**Figure 8:**
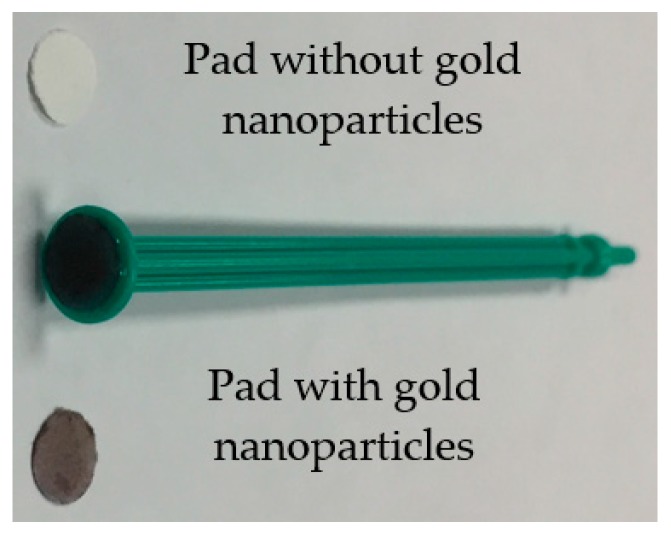
Photograph of the collection pads before and after immobilizing the gold nanoparticles, along with a rudimentary swabbing rod. The pads are ~5 mm in diameter.

The gold nanoparticles were synthesized as follows: A mixture of chloroauric acid and cetyltrimethyl-ammonium bromide, dissolved in 500 mL of HPLC-grade water, was brought to a boil in a 1000 mL round-bottom flask equipped with a condenser. A 100 mL solution of sodium citrate was added and refluxed to yield gold nanoparticles of ~20 nm in diameter. SERS pads were prepared by using a paper punch to create 5 mm diameter pads from the glass fiber sheets. These were immersed into the gold nanoparticle solution for 1 min, dried in an oven for 1 h at 37 °C, and allowed to stabilize overnight. The pads were then glued to a plunger from a 1 mL syringe to produce a rudimentary swabbing rod (Figure 8).

SERS measurements were performed using a 5 lb field-usable Raman spectrometer of in-house design that employed a 785 nm diode laser and a room temperature 1024 channel Si array detector that provided ~10 cm^−1^ resolution. An f/#2 lens produced an ~50 micron diameter spot on the sample, located 2 cm from the lens. The strips were inserted into a simple enclosure attached to the spectrometer that aligned the focal point of the laser on the SERS pad. All measurements used 40 mW laser power and a 3-sec acquisition time. All sample preparations were performed in a laboratory hood following standard safety precautions. The Raman spectra were part of a purchased library from Nicolet (Madison, WI, USA) that used ~500 mW at 1064 nm and an interferometer with a single element Ge detector that provided 4 cm^−1^ resolution.

## 4. Conclusions

A simple SERS-active pad was developed to measure opioids on surfaces. The quantitative and sensitive nature of these pads was demonstrated by successfully obtaining high-quality SERS of codeine and fentanyl in water from 50 µg/mL to 50 ng/mL, and from 50 µg/mL to 100 ng/mL, respectively. The pads were successfully used to measure 500 pg of fentanyl dried on a glass surface in ~5 min. These measurements compare quite favorably to a previous report of 500 ng fentanyl solutions deposited on epoxy and wood surfaces that were collected with filter paper impregnated with silver nanoparticles and measured [24]. These data strongly suggest that these pads, used with portable Raman analyzers, would be invaluable to airport security, drug raids, crime scenes, and forensic analysis.

## Supplementary Materials

The following are available online at https://www.mdpi.com/1420-3049/24/14/2578/s1. Figure S1: Photographs of 10 µL of 50 ng/mL fentanyl dried on a glass slide a) with a flash and b) without. Figure S2. Spectral average of three 10 µL samples of 50 ng/mL fentanyl dried on a glass slide that was used to determine the peak height, signal-to-noise ratio, and the limit of detection.
