# Supplementary material for: Quantitative Measurements of Codeine and Fentanyl on a Surface-Enhanced Raman-Active Pad
Source: Molecules. 2019 Jul 16;24(14):2578. doi: 10.3390/molecules24142578 (PMC6680930; doi:10.3390/molecules24142578)
Supplement: Supplementary file 1 [file molecules-24-02578-s001.pdf]

Supplemental Materials.

Figure S1 shows that a 10 µL of 50 ng/mL fentanyl dried on a glass slide is virtually invisible without flash photography.

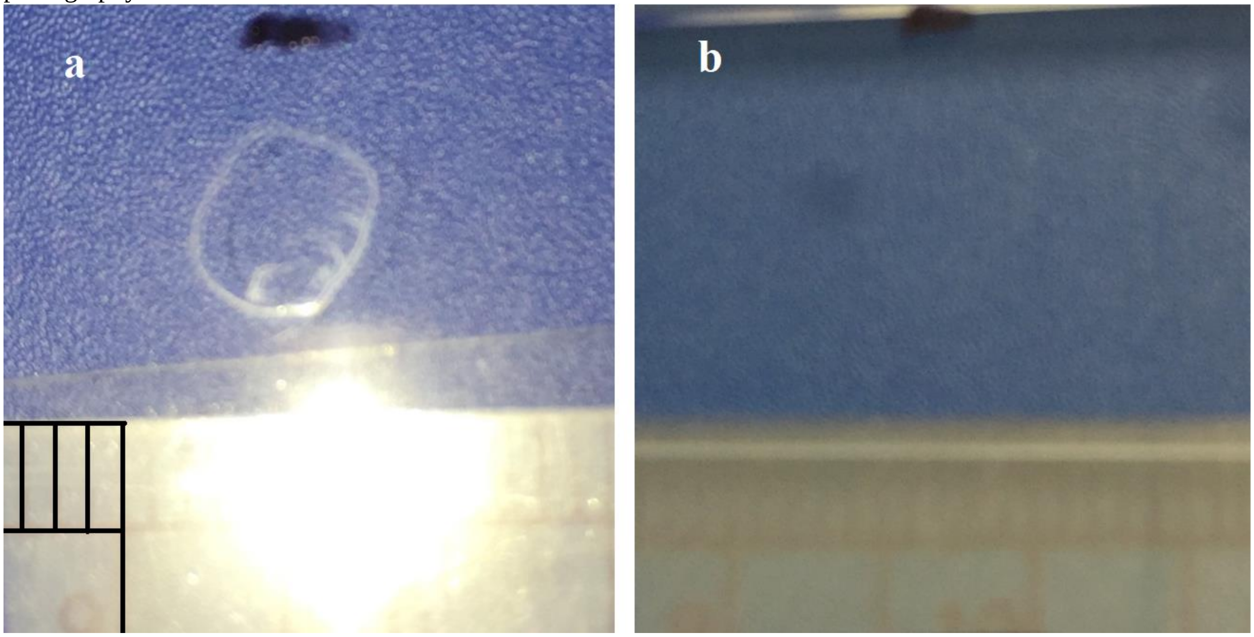

Figure S1. Photographs of 10 µL of 50 ng/mL fentanyl dried on a glass slide a) with a flash and b) without. The solvent ring is 4 to 5 mm in diameter (1 mm lines added for clarity). A shadow is apparent in a).

The spectra of the three 10 µL of 50 ng/mL fentanyl samples dried on a glass slide were used to estimate a limit of detection (LOD)for this experiment. Figure S2a shows an image of Real-Time Analyzers' software used to calculate the peak height and signal-to-noise ratio (S/N). The spectral width of the noise is chosen to match that of the spectral peak (signal). The peak heights for the three spectra were 384, 408, and 444, with an average of 412 with a standard deviation of 30 (7.3%). These samples each represent 500 pg of fentanyl, and based on a S/N LOD of 3, this suggest that ~40 pg could be detected ((500/(36.1/3)=41.6).

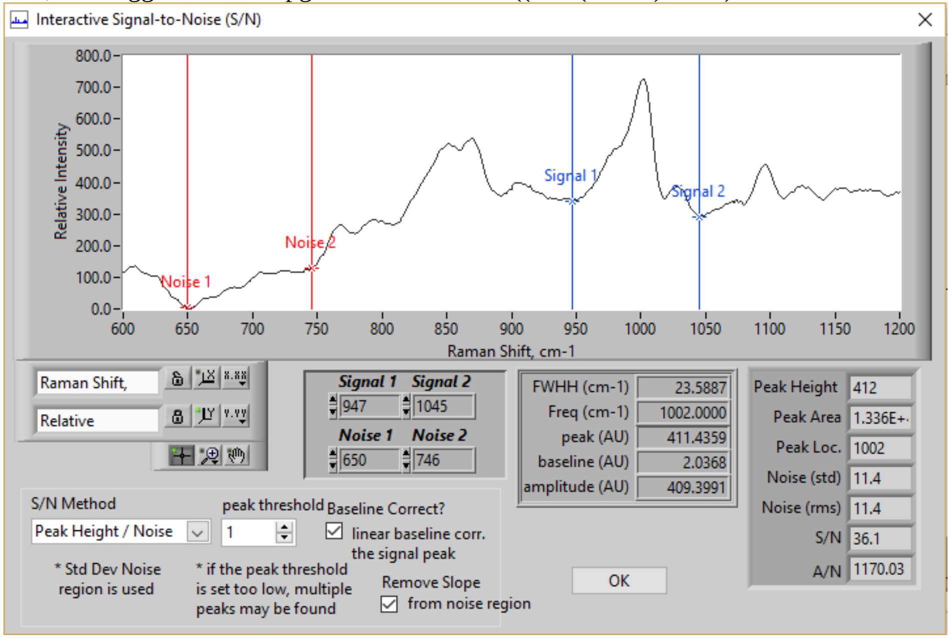

Figure S2. Spectral average of three 10 µL samples of 50 ng/mL fentanyl dried on a glass slide that was used to determine the peak height, signal-to-noise ratio, and the limit of detection. The baseline was set to 0 at 650 cm^{-1}.
